# Cryptosporidial Infection Suppresses Intestinal Epithelial Cell MAPK Signaling Impairing Host Anti-Parasitic Defense

**DOI:** 10.3390/microorganisms9010151

**Published:** 2021-01-12

**Authors:** Wei He, Juan Li, Ai-Yu Gong, Silu Deng, Min Li, Yang Wang, Nicholas W. Mathy, Yaoyu Feng, Lihua Xiao, Xian-Ming Chen

**Affiliations:** 1 Center for Emerging and Zoonotic Diseases, College of Veterinary Medicine, South China Agricultural University, Guangzhou 510642, China; hwhewei0707@163.com (W.H.); yyfeng@scau.edu.cn (Y.F.); lxiao@scau.edu.cn (L.X.); 2 Department of Medical Microbiology and Immunology, Creighton University School of Medicine, Omaha, NE 68198-5880, USA; lijuan413@126.com (J.L.); aiyugong@creighton.edu (A.-Y.G.); siludeng1@creighton.edu (S.D.); minli@creighton.edu (M.L.); yangwang@creighton.edu (Y.W.); nicholasmathy@creighton.edu (N.W.M.); 3 Institute of Animal Health, Guangdong Academy of Agricultural Sciences, Guangzhou 510640, China

**Keywords:** MAPK, p38/MAPK, *Cryptosporidium*, cryptosporidiosis, intestinal epithelium, defense

## Abstract

*Cryptosporidium* is a genus of *protozoan parasites* that infect the gastrointestinal epithelium of a variety of vertebrate hosts. Intestinal epithelial cells are the first line of defense and play a critical role in orchestrating host immunity against *Cryptosporidium* infection. To counteract host defense response, *Cryptosporidium* has developed strategies of immune evasion to promote parasitic replication and survival within epithelial cells, but the underlying mechanisms are largely unclear. Using various models of intestinal cryptosporidiosis, we found that *Cryptosporidium* infection caused suppression of mitogen-activated protein kinase (MAPK) signaling in infected murine intestinal epithelial cells. Whereas expression levels of most genes encoding the key components of the MAPK signaling pathway were not changed in infected intestinal epithelial cells, we detected a significant downregulation of *p38*/*Mapk*, MAP kinase-activated protein kinase 2 (*Mk2*), and *Mk3* genes in infected host cells. Suppression of MAPK signaling was associated with an impaired intestinal epithelial defense against *C. parvum* infection. Our data suggest that cryptosporidial infection may suppress intestinal epithelial cell MAPK signaling associated with the evasion of host antimicrobial defense.

## 1. Introduction

*Cryptosporidium*, a protozoan parasite that infects a variety of vertebrate hosts [1,2], can cause a life-threatening infection in the gastrointestinal tract and other mucosal surfaces in AIDS patients [3,4]. More recent epidemiological studies have revealed that *Cryptosporidium* is one of the most common pathogens responsible for moderate-to-severe diarrhea in children younger than two years old [5]. Human infections are mainly caused by two species: *C. parvum* and *C. hominis* [6]. *Cryptosporidium* oocysts undergo excystation and release infective sporozoites; the released sporozoite then attach to the apical membrane of intestinal epithelial cells and forms an intracellular vacuole in which the parasite develops [7]. The internalized sporozoite then matures and undergoes asexual reproduction (merogony) to produce merozoites and release into the lumen. Merozoites can then either infect other epithelial cells or mature into gametocytes (sexual reproduction). After fertilization in the intestinal tract, oocysts are generated and shed in the feces of an infected host.

Epithelial cells are an important component of gastrointestinal mucosal immunity [8]. They establish various types of barriers to protect the intestinal mucosa from commensal microbes or invasion of pathogenic organisms. Due to the intracellular but extracellular nature of the parasitophorous vacuole established by *Cryptosporidium* in infected host cells, epithelial cells play a critical role in the initiation, regulation, and resolution of both innate and adaptive immune reactions against *Cryptosporidium* infection [9]. Following *Cryptosporidium parvum* infection, intestinal epithelial cells display a series of early innate immune reactions, including expression of adhesion molecules, production and release of antimicrobial peptides and inflammatory chemokines and cytokines [10,11,12,13,14]. Production of antimicrobial peptides (e.g., β-defensin 2) and nitric oxide can kill *C. parvum* or inhibit parasite growth [15]. The release of chemokines and cytokines from infected epithelial cells can also mobilize and activate immune effector cells to the infection sites [9]. Activation of the *TLR*/*MyD88*/*NF-ĸB* signaling pathway appears to be essential for these epithelial responses [16].

To enable the completion of its life cycle in the host, *Cryptosporidium* has developed strategies of immune evasion within infected epithelial cells, particularly during the early infection stages [17,18]. However, the underlying mechanisms of immune evasion are largely unclear. *Cryptosporidium* infection can activate NF-κB signaling to activate anti-apoptotic cell death signaling in infected cells, which may benefit the parasite survival within infected epithelial cells [17]. The infection can cause the depletion of signal transducer and activator of transcription 1α (STAT1α), a critical transcription factor in IFN-γ signaling, resulting in the suppression of IFN-γ-dependent gene transactivation in intestinal epithelium [19]. The infection of host epithelial cells suppresses the expression of the C-C motif chemokine ligand 20 (CCL20), a cytokine with anti-parasitic capacity, which is detrimental to parasite clearance [20].

In this study, we present data by demonstrating suppression of the mitogen-activated protein kinase (MAPK) signaling in murine intestinal epithelium following *C. parvum* infection. Whereas expression levels of most genes coding the key components of the MAPK signaling pathway were not changed in infected intestinal epithelial cells, we detected significant downregulation of *p38*/*Mapk*, MAP kinase-activated protein kinase 2 (*Mk2*), and *Mk3* genes in infected host cells. Suppression of MAPK signaling was associated with an impaired intestinal epithelial defense against *C. parvum* infection. Our data suggest that cryptosporidial infection may suppress intestinal epithelial cell MAPK signaling to counteract host antimicrobial defense.

## 2. Materials and Methods

### 2.1. C. parvum and Cell Lines

*C. parvum* oocysts were purchased from a commercial source (Iowa strain, Bunch Grass Farm, Deary, ID, USA). The IEC4.1 cell line, transformed but non-tumorigenic *intestinal* epithelial cells from neonatal mice [21], was received as a kind gift from Dr. Pingchang Yang (McMaster University, Hamilton, ON, Canada). The muINTEPI, a murine intestinal epithelial cell line [22], was purchased from InSCREENeX Cellular Screening Technologies (Lower Saxony, Germany). Culture media were supplied with 10% FBS (Ambion, MA, USA) and antibiotics (100 IU/mL of penicillin and 100 µg/mL of streptomycin).

### 2.2. Infection Models and Infection Measurements

Models of intestinal cryptosporidiosis using intestinal epithelial cell lines were employed, as previously described [23,24]. The neonatal murine infection model of intestinal cryptosporidiosis was used for in vivo experiments [12,25]. Neonates (5 days after birth) received *C. parvum* oocysts by oral gavage (10^5^ oocysts per mice) to develop intestinal cryptosporidiosis. Mice received phosphate buffered saline (PBS) by oral gavage were used as control. At 24, 48, and 72 h after *C. parvum* oocysts or PBS administration, animals were sacrificed, and ileum intestine tissues were collected. At least five animals from each group were sacrificed and ileum epithelium tissues were obtained for biochemical analyses. Real-time PCR, immunofluorescence microscopy, and immunohistochemistry were used to assess *C. parvum* infection, as previously reported [24,26]. Anti-PCNA (Proliferating cell nuclear antigen, Abcam, MA, USA) was used to stain proliferating cells.

### 2.3. Agilent Microarray Analysis

The Agilent SurePrint G3 Human Gene Expression Microarray and service to process the samples were applied to genome-wide analysis, as previously described [15]. Briefly, cells were collected after exposure to *C. parvum* infection for 24 h. Total RNA was isolated using the RNeasy Mini kit (Qiagen, Hilden, Germany). A mixture of equal amounts of total RNAs from each group was used as the control. RNA (2 µg RNA) from each sample was labeled with the Agilent Gene Expression Hybridization Kit (Agilent, CA, USA). Hybridization and quantification of the labeled signals were preformed and the LC Sciences were carried out in accordance with MIAME guidelines.

### 2.4. Quantitative Real-Time PCR and Western Blot

For quantitative analysis of mRNA and *C. parvum* RNA expression, comparative real-time PCR was performed, as previous reported [15,23,24], using the SYBR Green PCR Master Mix (Applied Biosystems, Carlsbad, CA, USA). Briefly, total RNA was isolated and possible remaining DNA was removed using TRI-reagent, treated with DNA-free Kit (Ambion, MA, USA). Real-time PCR was then performed using 25 ng of template cDNA from reverse transcription for each RNA gene of interest. The expression level of each RNA was calculated using the ^ΔΔ^Ct method and normalized to glyceraldehyde-3-phosphate dehydrogenase (*Gapdh*). The sequence for all the PCR primers were listed in Table S1. For Western blotting, whole cell extracts were prepared using the Mammalian Protein Extraction Reagent (Fisher) with cocktail protease inhibitors. Cell pellet was incubated in the Mammalian Protein Extraction Reagent, centrifuged at 16,100× g for 20 min and the supernatants were collected. The following antibodies to Phospho-p38/Mapk (Cell Signaling Technology), p38/Mapk (Cell Signaling Technology) and Gapdh (Sigma-Aldrich, MO, USA, 0.2 µg/mL) were used. Details for Western blot were as described in our previous studies [15,23,24].

### 2.5. Statistical Analysis

All values are presented as mean ±S.E. Means of each group were from at least three independent experiments and compared with Student’s *t* test (unpaired) or the ANOVA test when appropriate. *p* values < 0.05 were treated as statistically significant.

## 3. Results

### 3.1. Suppression of MAPK Signaling in Intestinal Epithelium Following C. parvum Infection

**Figure 1:**
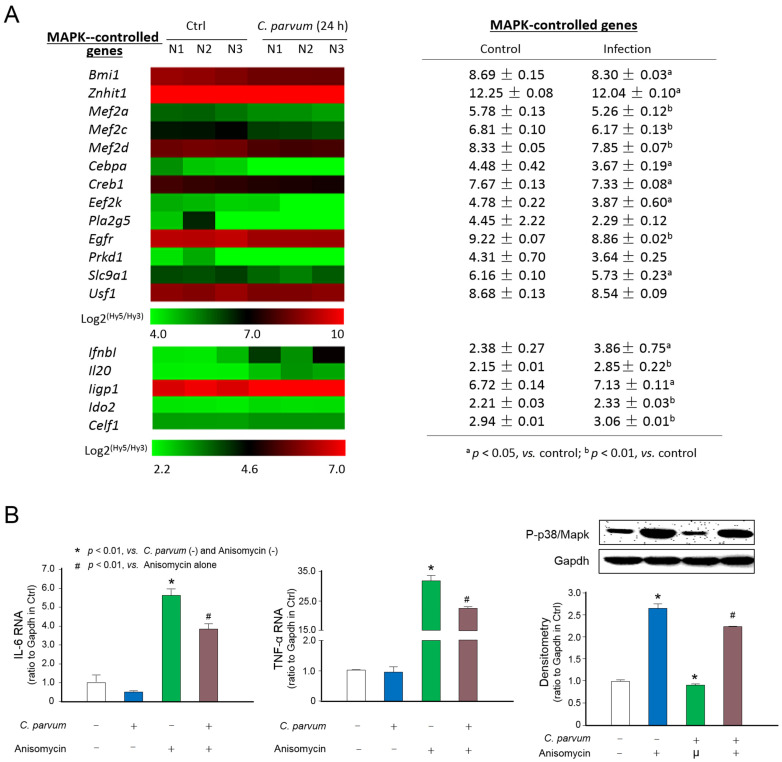
Suppression of MAPK signaling in intestinal epithelium following *C. parvum* infection. (**A**) Heatmap of expression levels of MAPK-controlled genes in host cells following *C. parvum* infection. IEC4.1 cells were exposed to *C. parvum* infection for 24 h. Total RNA was isolated for genome-wide transcriptome analysis via microarray. Expression levels of MAPK-controlled genes and selected inflammatory genes not directly related to the MAPK signaling are presented as the log2 (Hy5/Hy3), which passed the filtering criteria variation across the samples (*n* = 3). ^a^ *p* < 0.05, vs., control; ^b^ *p* < 0.01, vs. control. (**B**) Suppression of Il-6 and Tnf-α expression and inhibition of phosphorylation of p38/Mapk in *C. parvum*-infected intestinal epithelial cells in response to MAPK activator stimulation. IEC4.1 cells were exposed to *C. parvum* infection for 24 h and then treated with the MAPK activator anisomycin for up to 4 h. Anisomycin-mediated expression levels of IL-6 and TNF-α were measured. Phosphorylation of p38/Mapk was assessed using Western blot. Gapdh was also blotted for control. “+” and “−” represent cells treated with and without the according reagents, respectively. Representative gel images were shown. Data represent three independent experiments.

We recently performed a genome-wide transcriptome analysis of IEC4.1 cells following *C. parvum* infection for 24 h [24]. Infected IEC4.1 cells demonstrated a significant alteration in gene expression profile (GEO database: GSE112247) [24]. Intriguingly, expression levels of MAPK-controlled genes, such as *Mef2a*/*C*/*D*, *Znhit1*, *Bmi-1*, *Usf1*, *Creb*, *Pla2*, and *Mnk1*/*2* [27,28,29,30,31,32,33], were generally either not changed or decreased in infected cells, with a 24.6% to 54.1% decrease compared to that in the non-infected control cells (Figure 1A).Consistent with results from previous studies [9,15,23,24], many other inflammatory genes not directly related to the MAPK signaling were upregulated in the infected cells, such as *Ifnb1*, *IL20*, *Ligp1*, *Ido2*, and *Celf1* (Figure 1A). Therefore, we speculated that *C. parvum* infection may suppress p38/MAPK signaling activity in infected intestinal epithelial cells. To address this possibility, we infected IEC4.1 cells with *C. parvum* for 24 h and then measured the expression levels of MAPK-controlled genes in infected cells in response to a MAPK activator (anisomycin, AN) [34]. Expression levels of *Il-6* and *Tnf-α*, both of which are representative genes induced through the MAPK signaling upon AN stimulation [35,36], were significantly lower in infected cells than that in AN-treated non-infected cells (Figure 1B). Accordingly, activation of MAPK signaling in response to anisomycin stimulation, reflected by the phosphorylation of p38/Mapk [35,36], was partially inhibited in infected cells compared to that in the noninfected control cells (Figure 1B). Therefore, *C. parvum* infection results in the suppression of MAPK signaling activity in infected intestinal epithelial cells.

### 3.2. Expression Profile of Genes Encoding the Key Elements of the MAPK Signaling Pathway in Intestinal Epithelial Cells Following Infection

**Figure 2:**
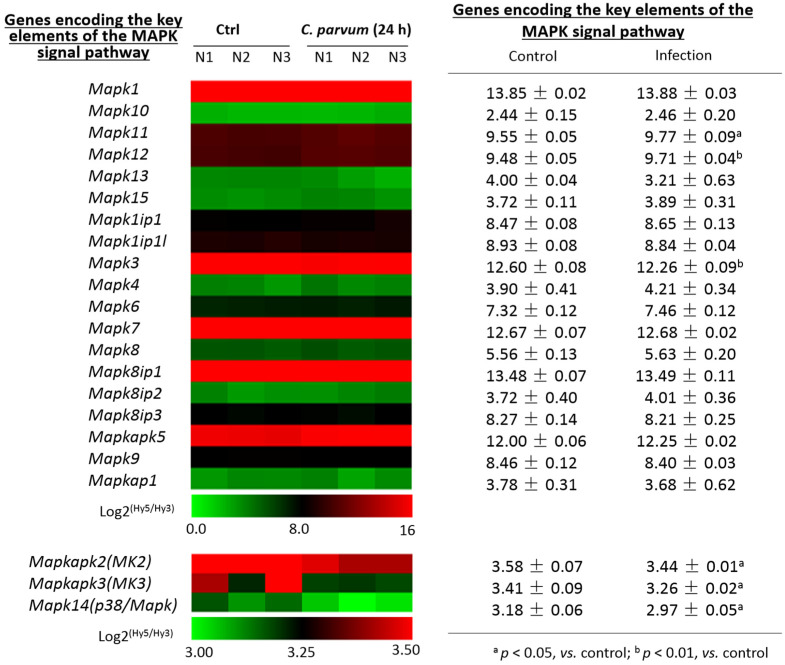
Expression profile of genes key to the MAPK signaling pathway in intestinal epithelial cells following *C. parvum* infection. Heatmap of expression levels of genes key to the MAPK signal pathway in host cells following *C. parvum* infection, presented as the log2 (Hy5/Hy3) ratios, which passed the filtering criteria variation across the samples (*n* = 3). IEC4.1 cells were exposed to *C. parvum* infection for 24 h and RNA was isolated for genome-wide transcriptome analysis via microarray. Expression levels of genes key to the MAPK signal pathway are shown. ^a^ *p* < 0.05, vs. control; ^b^ *p* < 0.01, vs. control.

Using the same dataset from our previous transcriptome analysis of IEC4.1 cells following infection by *C. parvum* for 24 h, as described above [24], we looked at the expression levels for these genes encoding the key elements of the MAPK signal pathway. Expression levels of most genes coding the key elements of the MAPK signaling pathway were not altered IEC4.1 in cells following *C. parvum* infection for 24 h (Figure 2). However, the expression levels of *p38*/*Mapk*, MAP kinase-activated protein kinase 2 (*Mk2*), and *Mk3* genes were significantly decreased in infected cells, compared to that in the non-infected control (Figure 2).

### 3.3. Downregulation of p38MAPK, MK2 and MK3 Genes in Infected Host Cells of Various Models of C. parvum Infection

**Figure 3:**
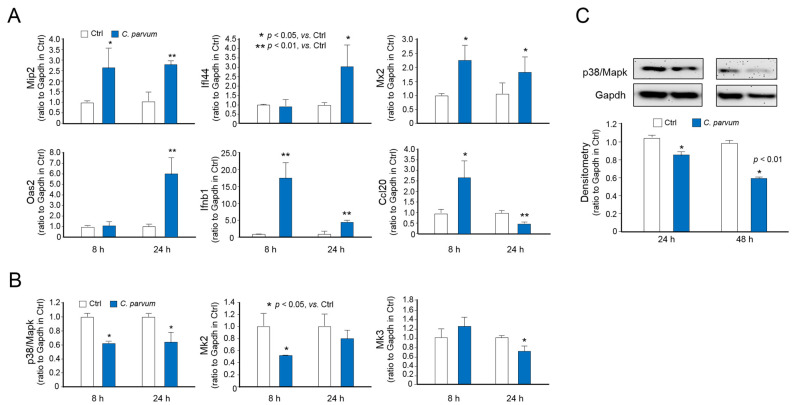
Downregulation of *p38*/*Mapk*, *Mk2* and *Mk3* genes in intestinal epithelial cells following *C. parvum* infection. (**A**) RNA levels of a panel of inflammatory genes in IEC4.1 cells following *C. parvum* infection. Cells were exposed to *C. parvum* infection for 8 and 24 h. RNA levels of these genes were measured by using real-time quantitative PCR. (**B**) RNA levels of *p38*/*Mapk*, *Mk2* and *Mk3* genes in IEC4.1 cells following *C. parvum* infection. Cells were exposed to *C. parvum* infection for 8 and 24 h. RNA levels of *p38*/*Mapk*, *Mk2* and *Mk3* genes were measured. (**C**) Protein level of p38/Mapk in IEC4.1 cells following *C. parvum* infection. Cells were exposed to *C. parvum* infection for 24 h and 48 h. Protein level of p38/Mapk was assessed by using Western blot. Gapdh was also blotted for control. Data represent three independent experiments.

Consistent with results from previous studies [24,37], we detected the upregulation of several inflammatory and defense genes using real-time PCR in IEC4.1 cells following *C. parvum* infection, including *Mip2*, *Ifi44*, *Mx2*, *Oas2*, and *Ifnb1* (Figure 3A). Whereas the induction of *Ccl20* was observed in IEC4.1 cells following *C. parvum* infection for 8 h, a significant decrease in the *Ccl20* expression level was detected in cells following infection for 24 h (Figure 3A), confirming the suppression of *Ccl20* in host cells following *C. parvum* infection in a previous report [20]. We further confirmed the decreased expression of *p38*/*Mapk*, *Mk2*, and *Mk3* in infected cells (Figure 3B). Moreover, Western blotting further confirmed decreased p38/Mapk protein content in infected IEC4.1 cells (Figure 3C).

**Figure 4:**
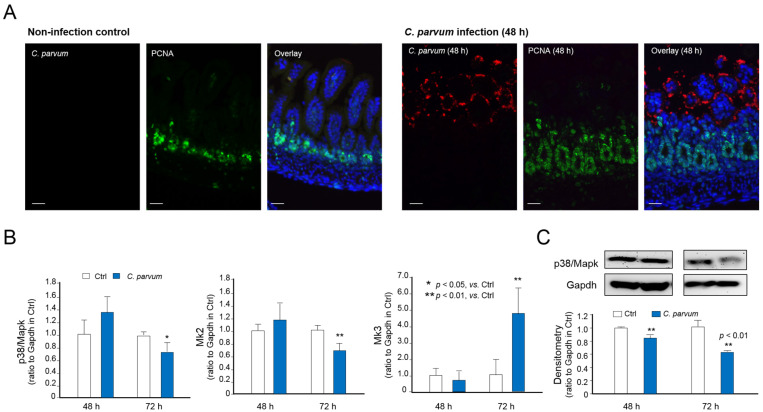
Downregulation of *p38*/*Mapk*, *Mk2* and *Mk3* genes in intestinal epithelium of neonatal mice following *C. parvum* infection in vivo. (**A**) Immunofluorescent staining of ileum from neonatal mice with and with *C. parvum* infection. Mice at the age of 5 days after birth received *C. parvum* oocysts by oral gavage (10^5^ oocysts each mouse). Mice which received phosphate buffered saline by oral gavage were used as control. Tissue sections were triple stained with anti-*C. parvum* (showing in red), anti-PCNA (showing proliferating cells in green) and DAPI (showing nuclei in blue). (**B**) RNA levels of *p38*/*Mapk*, *Mk2* and *Mk3* genes in isolated intestinal epithelium from infected neonatal animals. RNA levels of *p38*/*Mapk*, *Mk2* and *Mk3* genes were measured by using real-time PCR. (**C**) Protein level of p38/Mapk in isolated intestinal epithelium from infected neonatal animals. Protein level of p38/Mapk was assessed by using Western blot. Gapdh was also blotted for control. DAPI = 4′,6-diamidino-2-phenylindole; PCNA = Proliferating cell nuclear antigen.

**Figure 5:**
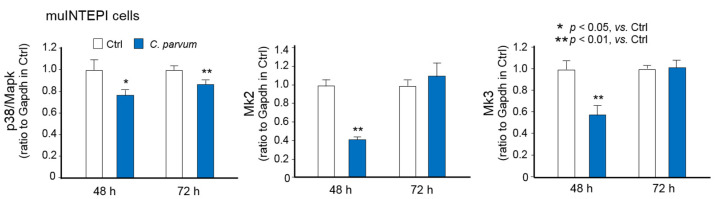
Downregulation of *p38*/*Mapk*, *Mk2* and *Mk3* genes in mulINTEP1 cells following *C. parvum* infection. RNA levels of *p38*/*Mapk*, *Mk2* and *Mk3* genes in mulINTEP1 cells following *C. parvum* infection. Cells were exposed to *C. parvum* infection for 48 and 72 h. RNA levels of *p38*/*Mapk*, *Mk2* and *Mk3* genes were measured by using real-time PCR. Data represent three independent experiments.

Using a well-documented model of intestinal cryptosporidiosis in neonatal mice through the oral administration of the parasite [12,25], we detected the infection of *C. parvum* to the intestinal epithelium at the villus region and increased cell proliferation at the crypt region, as previously reported [12,25], by immunofluorescent staining (Figure 4A). We also detected the decreased expression of *p38*/*Mapk*, *Mk2*, and *Mk3* in isolated intestinal epithelium from infected neonatal mice (Figure 4B). Western blotting further confirmed a decrease of p38/Mapk protein content in the intestinal epithelium from infected neonatal mice (Figure 4C). In addition, decreased expression of *p38*/*Mapk*, *Mk2*, and *Mk3* genes in infected cells was detected in another murine intestinal epithelial cell line (mulINTEP1 cells) following *C. parvum* infection [22] (Figure 5).

### 3.4. Suppression of MAPK Signaling Impairs Intestinal Epithelial Innate Defense against C. parvum

**Figure 6:**
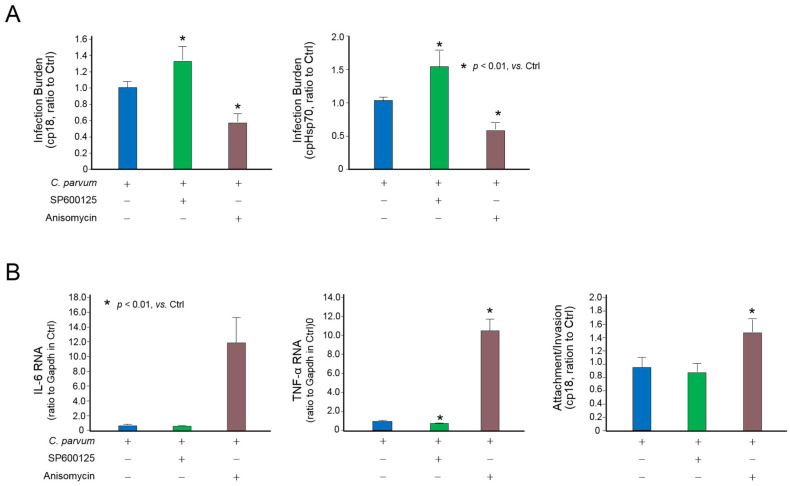
Suppression of MAPK signaling impairs intestinal epithelial defense against *C. parvum* infection. (**A**) Activation of MAPK signaling decreased the infection burden of *C. parvum* in host cells. IEC4.1 cells were exposed to *C. parvum* infection for 24 h in the presence or absence of the MAPK activator anisomycin or inhibitor SP600125. Infection burden of *C. parvum* was quantified. (**B**) Suppression of MAPK signaling in intestinal epithelial cells on the attachment and invasion of *C. parvum* to host cells. Cells were exposed to *C. parvum* infection for 2 h (for attachment and invasion) in the presence or absence of the MAPK activator anisomycin or inhibitor SP600125. Infection burden of *C. parvum* was quantified. The expression levels of IL-6 and TNF- were measured in cells treated with anisomycin or SP600125 to confirm their effects on MAPK signaling. “+” and “−” represent cells treated with and without the according reagents, respectively. Data represent three independent experiments.

We then investigated the possible impact of the suppression of MAPK signaling on host epithelial antimicrobial defense. When cells were exposed to *C. parvum* oocysts for infection in the presence of a MAPK activator anisomycin, a significant decrease of infection burden was detected (Figure 6A). Accordingly, an increase of infection burden was observed in cells treated with a MAPK inhibitor, SP600125 [38] (Figure 6A). The impact of MAPK signaling on the infection burden in IEC4.1 cells was not due to the effects on parasite attachment or invasion, as a similar attachment/invasion pattern was observed in cells treated with the MAPK inhibitor SP600125 (Figure 6B). Interestingly, a slight increase of the parasite attachment/invasion was measured in cells treated with the MAPK activator anisomycin (Figure 6B), suggesting that the alterations in the infection burden (after exposure to parasites for 24 h as measured in Figure 6A) in cells treated with the MAPK activator or inhibitor was due to MAPK-mediated epithelial anti-parasite defense, rather than their impact on the parasite attachment or invasion.

## 4. Discussion

Several immune-evasive strategies have been proposed to address how *C. parvum* evades host innate antimicrobial defense [39]. As one of the ancient host antimicrobial strategies to intracellular pathogens [40], the anti-apoptotic mechanism in infected host cells during early *C. parvum* infection stage may facilitate parasite propagation and survival [17,41]. Whereas *C. parvum* infection eventually causes host cell death, infection inhibits apoptotic cell death of directly infected host cells during the early stage of infection [41]. Moreover, it has been demonstrated that infection induces depletion of STAT1α and suppresses expression of CCL20 in intestinal epithelial cells, resulting in the suppression of epithelial antimicrobial defense [13,20]. In this study, we observed the suppression of p38/MAPK signaling in intestinal epithelium following *C. parvum* infection. We detected significant downregulation of *p38*/*Mapk*, *Mk2*, and *Mk3* genes in infected intestinal epithelial cells. Furthermore, suppression of p38/MAPK signaling was associated with an impaired intestinal epithelial defense against *C. parvum* infection. Therefore, our data suggest a new strategy by which *C. parvum* impedes host antimicrobial signaling to evade host immune defense.

Intestinal epithelial cells express several pathogen pattern recognition receptors, such as Toll-like receptors (TLRs), C-type lectin receptors, NOD-like receptors and RIG-I-like receptors. While their receptor-proximal signaling mechanisms vary, these pattern recognition receptors can activate both MAPK and NF-κB pathways, which are crucial to generating immune responses [42]. We and other laboratories previously demonstrated activation of the TLR/MyD88/NF-κB signal pathway in intestinal epithelium following *Cryptosporidium* infection [16,17,41]. Nevertheless, here we observed that the vast majority of MAPK-controlled genes show either no change or are suppressed in intestinal epithelial cells following *C. parvum* infection. Suppression of MAPK signaling in infected cells was evident by a significant inhibition of cellular responses to anisomycin stimulation. How *Cryptosporidium* infection impedes MAPK signaling activation is still unclear. Nevertheless, we detected significant downregulation of *p38*/*Mapk*, *Mk2* and *Mk3* in infected cells. Consistent with results from a previous report [20], we also detected downregulation of Ccl20 in host cells following *C. parvum* infection for 24 h. Of note, Ccl20 has been demonstrated as one of the defense genes that are controlled by MAPK and NF-κB signaling in intestinal epithelial cells [35,36,43,44].

The expression of proteases that can degrade specific components of MAPK cascades is one mechanism used by pathogens to modulate MAPK signaling in infected host cells. For examples, the leishmanolysin (also known as GP63) protease of *Leishmania major can* degrade TGF-beta activated kinase 1/MAP3K7 binding protein 1, resulting in the inhibition of MAPK7-mediated p38α activation in infected host cells [45]. The lethal toxin from *Bacillus anthracis* contains a protease, which can cleave the MAPK-docking domain of MKKs, and thus, inhibits MAPK activation and suppresses inflammatory cytokine production in macrophages [46]. Similarly, several members of the *Yersinia* bacterial genus use type III secretion systems to inject proteins into host cells to interfere with host intracellular signaling [47]. YopJ is one of such proteins that can inhibit both MAPK and NF-κB signaling in host cells (40). Interestingly, *Cryptosporidium* species carry *Cryptosporidium parvum virus 1* (CSpV1) virus, a virus of the family *Partitiviridae*, genus *Cryspovirus* that infects *Cryptosporidium* (41). The CSpV1 genome comprises two distinct dsRNAs, sized 1786 bp (CSpV1-dsRdRp) and 1374 bp (CSpV1-dsCA) (42). The predicted protein sequence from the CSpV1-dsCA has a limited similarity with mitogen-activated c-June NH2 terminal protein kinases (JNK/p38/MAPK) from mammalian cells (42). It merits further investigation to clarify whether CSpV1 dsRNAs from *Cryptosporidium* can target MAPK signaling in infected host cells. RNA transcripts with very low protein-coding potential from *Cryptosporidium* have been demonstrated to be delivered into infected host epithelial cells and modulate host cell gene transcription [26].

Pathogens have often evolved effective mechanisms of inhibiting immune responses and a common strategy is targeting the host intracellular signaling networks, including the NF-κB and interferon signal pathways [48]. Given the importance of MAPK signaling in regulating the immune response, it is not surprising that many pathogens can modulate MAPK activation to evade host immune defense, such as *Bacillus anthracis*, *Leishmania major*, and *M. tuberculosis* [45,46,47,49]. Coupled with the fact that the MAPK signaling pathway usually cross-talks with many other signaling cascades important to epithelial innate defense [42], we would speculate that the suppression of MAPK signaling is associated with an impaired intestinal epithelial immunity against *Cryptosporidium* infection. Our findings also suggest the potential of the pharmacological targeting of MAPK signaling to control *Cryptosporidium* infection.

## Data Availability

Publicly available datasets were analyzed in this study. This data can be found here: https://www.ncbi.nlm.nih.gov/geo/query/acc.cgi?acc=GSE112247.

## Supplementary Materials

The following are available online at https://www.mdpi.com/2076-2607/9/1/151/s1, Table S1: List of primers used for RT-PCR.
